# 30 Days in the Life: Daily Nutrient Balancing in a Wild Chacma Baboon

**DOI:** 10.1371/journal.pone.0070383

**Published:** 2013-07-24

**Authors:** Caley A. Johnson, David Raubenheimer, Jessica M. Rothman, David Clarke, Larissa Swedell

**Affiliations:** 1 Doctoral Program in Anthropology, The Graduate Center, City University of New York, New York, New York, United States of America; 2 New York Consortium in Evolutionary Primatology, New York, New York, United States of America; 3 Department of Anthropology, Queens College of the City University of New York, Queens, New York, United States of America; 4 Department of Archaeology, University of Cape Town, Cape Town, South Africa; 5 Charles Perkins Centre, Faculty of Veterinary Science and School of Biological Sciences, The University of Sydney, Sydney, New South Wales, Australia; 6 Department of Anthropology, Hunter College of the City University of New York, New York, New York, United States of America; 7 Department of Biology, Queens College of the City University of New York, Queens, New York, United States of America; Max Planck Institute for Evolutionary Anthropology, Germany

## Abstract

For most animals, the ability to regulate intake of specific nutrients is vital to fitness. Recent studies have demonstrated nutrient regulation in nonhuman primates over periods of one observation day, though studies of humans indicate that such regulation extends to longer time frames. Little is known about longer-term regulation in nonhuman primates, however, due to the challenges of multiple-day focal follows. Here we present the first detailed study of nutrient intake across multiple days in a wild nonhuman primate. We conducted 30 consecutive all day follows on one female chacma baboon (*Papio hamadryas ursinus*) in the Cape Peninsula of South Africa. We documented dietary composition, compared the nutritional contribution of natural and human-derived foods to the diet, and quantified nutrient intake using the geometric framework of nutrition. Our focus on a single subject over consecutive days allowed us to examine daily dietary regulation within an individual over time. While the amounts varied daily, our subject maintained a strikingly consistent balance of protein to non-protein (fat and carbohydrate) energy across the month. Human-derived foods, while contributing a minority of the diet, were higher in fat and lower in fiber than naturally-derived foods. Our results demonstrate nutrient regulation on a daily basis in our subject, and demonstrate that she was able to maintain a diet with a constant proportional protein content despite wide variation in the composition of component foods. From a methodological perspective, the results of this study suggest that nutrient intake is best estimated over at least an entire day, with longer-term regulatory patterns (e.g., during development and reproduction) possibly requiring even longer sampling. From a management and conservation perspective, it is notable that nearly half the subject’s daily energy intake derived from exotic foods, including those currently being eradicated from the study area for replacement by indigenous vegetation.

## Introduction

Food choices and associated variation in nutrient and energy intake are the primary determinants of growth patterns, survival, and fertility, and are directly linked to lifetime reproductive success [1]. Traditionally these relationships have been modeled as a process of maximizing the gain of a particular limiting resource, most commonly energy [2], or single nutrients such as protein [3]. A large number of recent studies, however, have demonstrated the utility of models that emphasize the interactive effects of nutrients on animals (reviewed in Simpson & Raubenheimer [4]). The geometric framework of nutrition (GF) was developed to quantify these interactive effects [5], [6].

From the nutritional geometry perspective, the expectation is that the primary nutritional goal of foraging should be to obtain nutrients in sufficient quantities and balanced proportions, not to maximize the gain of any single food component. When available foods prevent the animal from achieving the target intake, then it faces a trade-off between over-ingesting some nutrients and under-ingesting others. In this circumstance the animal is expected to select a diet that provides the combination of nutrient surpluses and deficits that minimizes the cost of its predicament. Studies in controlled laboratory settings have used nutritional geometry to demonstrate that animals do regulate to an intake target [7], [8], that the target diet provides fitness benefits [9], and that when constrained from obtaining the balanced diet animals show a distinctive pattern of trade-off that minimizes the cost of nutritional imbalance [10].

Most recently, the nutritional geometry approach has been applied to field studies of nonhuman primates. For example, Felton et al. [11] measured across an annual cycle the daily food intakes of Peruvian spider monkeys (*Ateles chamek*) in a Bolivian rainforest. Results showed that the monkeys targeted a non-protein energy (NPE) to protein (P) balance of approximately 8∶1 kcal, and when available foods constrained them from achieving this balance they relaxed regulation of NPE and prioritized P. Similarly, Rothman et al. [12] used nutritional geometry to analyze relationships between available protein and non-protein energy in mountain gorillas (*Gorilla beringei*), which were found to target a diet with a NPE:P ratio of 3∶1 kcal. When the composition of available foods prevented them from achieving this balance, they showed the opposite pattern from spider monkeys, namely over-eating protein to maintain a constant NPE intake. Like spider monkeys, humans prioritize P intake and will thus over eat fats, carbohydrates and energy when protein density in the diet is low [13]–[15]. Simpson et al. [16] proposed that this regulatory pattern has interacted with the reduced proportion of protein in the modern human diet to drive an increase in energy intake and obesity.

Studies of nutritional geometry in wild primates to date have used all-day focal follows with continuous behavioral sampling on multiple focal subjects in which repeat observations of the same individual usually occur on non-consecutive days [12], [17]. This brings the apparent advantage of a larger sample size but limits the scope of study to nutrient regulation over a maximum of one day. However, evidence that feeding regulation occurs over multi-day timescales in at least one primate, humans [18], raises the question of how nutrient intake fluctuates over timescales greater than one day in free-ranging nonhuman primates. To answer this question, we take the complementary approach of focusing our effort on a single individual for a 30-day period in order to examine daily nutrient regulation over an entire month, thereby providing unprecedented detail regarding the timescale of nutritional regulation.

A high dietary diversity characterizes chacma baboons (*Papio hamadryas ursinus*) throughout their range, from the Cape Peninsula in the south to the deserts of Namibia and the Okavango delta in the north [19]–[26]. Studies of nutrient composition have suggested that chacma baboons select foods that are high in protein and low in fiber, total phenolics and alkaloids [21], [26]. The diet of Cape Peninsula baboons has been shown to consist largely of grasses, underground storage organs, and pine nuts [25], [27], [28]. Most baboons in the Cape Peninsula also consume human-derived foods through provisioning, crop raiding, and access to discarded foods in garbage bins [25], [27], [29]. While the chacma baboon as a species is not threatened, the Cape Peninsula population experiences high levels of anthropogenic injury and mortality and has received protected status [30]. Moreover, the Cape Peninsula population is effectively isolated from other baboon populations due to urban sprawl, thereby impeding male dispersal and increasing baboon-human conflict [28], [31], [32]. Approximately one third of the Cape Peninsula baboon population uses the Tokai Forest; this forest is currently being harvested and returned to the indigenous fynbos vegetation, a dry-adapted locally endemic floral community [28], [33]. Despite this impending land transformation and its possible impact on the baboons in Tokai, the feeding ecology and nutritional intake of this population are still poorly understood [28], [31].

Our goal here was to determine how the chacma baboon manages its nutrient intake in this altered environment. Specifically, we are testing: (i) whether it is able to regulate a macronutritional intake target; (ii) if constrained from regulating to an intake target, how it resolves the conflicting demands for different macronutrients; (iii) over what timescale it regulates macronutrient intake, whether to a target or a constrained compromise; and (iv) what role human-derived foods play in its nutritional strategy. This should enable us to test for a mismatch between baboon nutritional biology and the changing environment of the Tokai Forest. Additionally, these results will expand the comparative database for understanding the ecology and evolution of macronutritional regulation in free-ranging primates.

## Methods

### Study Site and Subject

This study took place from 4 July 2010 to 3 August 2010 in the Tokai Forest (34°03′17 S, 18°23′59 E) of the Table Mountain National Park (TMNP) 20 km south of Cape Town, South Africa. The Cape Peninsula is characterized by a Mediterranean climate with warm dry summers and cool wet winters [33]. The Tokai section of the TMNP includes commercial plantations dominated by exotic species such as *Pinus* sp. and *Eucalyptus* sp. as well as areas of indigenous fynbos vegetation, a dry-adapted locally endemic floral community [33]. Bordering the national park land are private vineyards and other agricultural land, as well as urban and suburban areas with concentrated human-derived food resources from garbage bins, gardens and fruit trees. At the time of this study, the study troop, MT1, consisted of approximately 65 individuals, including 20 adult females and 5 adult males. The troop’s home range lies mainly within the national park land, but the baboons commonly forage on human-derived foods at the interface between the park, private land, and public spaces. They are herded away from the urban edge and into the park by “baboon monitors” employed by the City of Cape Town. The baboons are thus well accustomed to human presence: they move towards humans that are carrying food (as a foraging strategy leading to baboon-human conflict), they move away from baboon monitors (who use aversive conditioning to herd them), and they remain neutral around researchers, who neither feed them nor herd them. We performed consecutive all day follows (n = 30) on a single adult female, STE, from the MT1 troop. STE was a middle ranking female who was neither pregnant nor lactating at the time of the study.

### Feeding Observations

We began our focal follows when the subject exited her sleeping tree in the morning and continued until she entered her sleeping tree at night (approx. 8∶00 to 18∶00). During focal follows, we recorded all foods consumed, noted the food species and plant part, and estimated the intake of food items by counting all plant parts eaten. We also recorded time spent feeding and performing other activities. We followed the subject for 275 hours across 30 days. The 30 days were almost entirely consecutive. On day 16, we failed to follow our subject, but we resumed focals on day 17 and continued for 14 days thereafter.

### Food Collection and Analysis

We collected samples of every naturally occurring food item consumed by our study subject. Following feeding bouts, we collected food items eaten from the same location as feeding events. We processed these foods in the same way that the study subject processed them. We weighed and dried (<40°C out of sunlight) samples at the field site and milled them in the Department of Botany at the University of Cape Town. Samples were then exported to the Nutritional Ecology Laboratory at Hunter College in New York, USA. All nutrient concentrations were estimated on a dry matter basis [34]. We measured hemicellulose and cellulose via neutral (NDF) and acid detergent fiber (ADF), and lignin via acid detergent lignin (ADL) [35], [36]. We measured crude protein through combustion [37] and available protein (AP) through subtraction of acid detergent insoluble nitrogen [38], [39]. We measured ash through combustion [34] and crude fat using ether extract [34]. We measured the percentage non-structural carbohydrates by difference, subtracting the contributions of NDF, crude protein, fat, and ash from the rest of the nutrients [34]. We estimated metabolizable energy using an equation by taking into account fiber digestibility [40]. To estimate fiber digestibility we collected fecal samples from the study subject and calculated a digestibility coefficient (18% NDF digestibility with 34% NDF in overall diet) using lignin as fecal marker as per Rothman et al. [41]. We defined human derived foods as those procured from anthropogenic sources such as garbage bins, gardens, and individual people. We obtained nutrition information for human-derived foods from the USDA nutrition database [42] or from product manufacturers. Plant samples were identified by Terry Trinder-Smith at the Bolus Herbarium of the University of Cape Town or Ernst van Jaarsveld at the Kirstenbosch National Botanical Garden of the South African National Biodiversity Institute, where voucher specimens are available.

### Data Analysis

To determine nutritional priorities we used the geometric framework of nutrition, a state-space modeling approach, in which we plotted the daily intake of nutrients and non-nutrient items (i.e. indigestible fiber) in graphical space, with each axis representing a different nutrient item in the diet [43], [44]. We plotted the daily contribution of non-protein (fats, carbohydrates) energy vs. available protein to the diet to determine patterns of nutrient prioritization. From this, we observed which parameters the subject allowed to vary when faced with variable food choices, and which diet components were maintained or prioritized. We used linear regression and coefficient of determination (R^2^) values to determine the balance of macronutrients ingested by the subject, and the relationship and strength (correlation) of that balance across days [12]. We also calculated coefficient of variation (CV) values for STE’s daily nutrient intakes. CV values close to zero demonstrate that the intake of that nutrient item is more constrained compared to those where CV values are large or greater than one.

We plotted right-angled mixture triangles (RMTs) to observe the relative daily contribution of available protein (AP), carbohydrates (non-structural carbohydrates and digestible fiber) (C) and fat (F) to the diet, and compared this to the AP, C, and F composition of foods to determine how patterns of intake related to food composition [45]. We used a two-tailed t-test to assess whether the slope of STE’s macronutrient intake differed from one, showing relatively constant protein intake compared to fat and carbohydrate intake. To address the long-term integrated outcome of daily food selection for STE, we plotted the cumulative NPE vs. AP intake across the 30-day study period.

For some of the observation time our subject was seen to be feeding but we were unable to observe her closely enough to estimate food intake (usually because she was feeding on private property). For this missed time (∼30% of feeding time), we estimated her total food intake by extrapolating her rate of intake during observed feeding sessions in similar locations to the missed observation time.

### Ethics Statement

Data for this study derived exclusively from behavioral observations and plant samples, and no contact with the study subjects occurred. All protocols for the fieldwork portion of this study were approved by the Queens College Institutional Animal Care and Use Committee (protocol #132). Permission was granted by South African National Parks (to L.S.) to conduct this research in the Tokai section of the Table Mountain National Park, and permits to export plant samples were granted (to L.S.) by CapeNature, the Western Cape regional biodiversity and conservation authority.

## Results

The focal subject consumed 69 naturally occurring food items from 29 species over the study period, represented by 80 food samples (see Table 1 for the staple diet). The naturally derived diet was composed of 58% herbaceous leaves, 15% mushrooms, 15% nuts and seeds, 9% underground storage organs, 2% fruits, and 3% flowers, lichens, stems and other minor items. In addition to the naturally derived foods, the subject’s diet also included 16 human derived food items, which together comprised 7% (dry weight) of the total diet. Human derived foods contributed 8% of total dietary energy intake, while exotic plant species (included in “natural” foods category) such as pine nuts (*Pinus pinea*) and acorns (*Quercus* sp.) contributed 44% of the total energy intake. The subject’s average wet mass intake of food per day was 1.9±0.8 kg.

**Table 1:** Nutritional composition of staple foods expressed on a dry matter basis (>1% of dry mass intake during study period).

Species	part	NDF	ADF	ADL	AP	Fat	Ash	TNC	E contrb.
*Arctotheca calendula*	leaf	33.49%	27.74%	8.12%	18.47%	2.42%	20.40%	25.22%	0.55%
*Bromus sp.*	leaf	54.05%	26.73%	2.09%	15.33%	3.89%	10.94%	15.79%	0.85%
*Cotula turbinata*	leaf	39.13%	29.96%	6.76%	8.69%	2.42%	7.71%	42.05%	0.79%
*Citrus sinensis* 1	peel	10.60%	×	×	1.50%	20.00%	×	25.00%	1.67%
*Malva sp.*	leaf	42.80%	32.53%	20.21%	12.02%	3.68%	20.95%	20.55%	1.19%
*Musa spp.* 2	peel	8.06%	×	×	10.00%	5.00%	×	20.00%	0.37%
*Ornithopus compressus*	leaf	41.16%	32.21%	16.36%	14.52%	3.93%	17.67%	22.72%	1.75%
*Oxalis luteola*	bulb	27.04%	23.05%	4.97%	6.25%	1.84%	7.35%	59.11%	1.49%
*Oxalis pes-caprae*	leaf	27.46%	21.54%	3.95%	12.88%	4.10%	11.30%	44.26%	3.69%
*Pinus pinea* 1	seed	3.70%	×	×	13.69%	68.37%	×	13.08%	29.31%
*Quercus sp.* 1	seed	×	×	×	6.15%	23.86%	×	40.75%	14.67%
*Tolpis capensis*	leaf	33.98%	24.06%	8.44%	11.24%	5.96%	20.49%	27.46%	15.11%
*Tolpis capensis*	root	32.47%	27.62%	13.91%	5.03%	2.07%	16.12%	44.76%	3.49%
*Trachyandra sp.*	leaf	39.23%	25.63%	2.55%	12.84%	4.31%	17.03%	26.59%	0.53%
*Unknown leaf*	leaf	41.38%	32.45%	19.84%	11.77%	1.37%	21.35%	24.13%	0.62%
*Vicia tetrasperma*	leaf	42.68%	31.60%	8.51%	16.90%	3.86%	7.01%	29.55%	4.74%

NDF = neutral detergent fiber, ADF = acid detergent fiber, ADL = acid detergent lignin, AP = available protein, TNC = total non-structural carbohydrates, E contrb. = contribution of food item to total metabolizable energy intake over the 30 day study period.

1 nutritional information from U.S. Department of Agriculture [42].

2 nutritional information from Emaga et al. [57].

**Figure 1:**
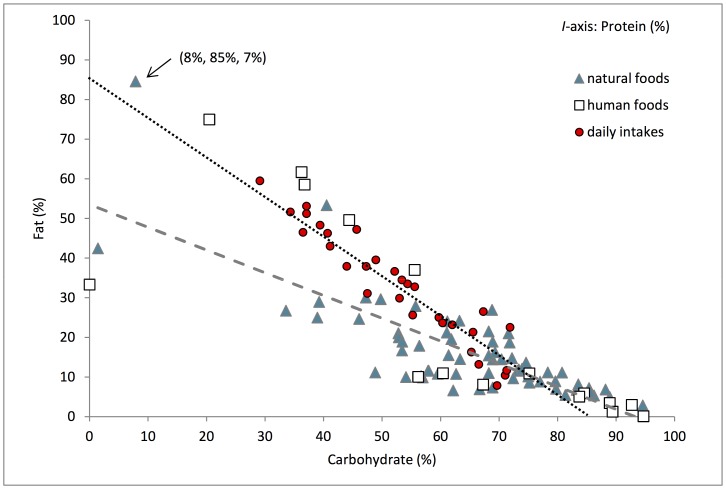
Relative contributions of fat, carbohydrate, and available protein to metabolizable energy intake and foods consumed. Right-angled mixture triangle (RMT) showing the relative contributions of fat vs. carbohydrate vs. available protein to (a) metabolizable energy intake for days 1–30 ( = circles), and to (b) foods (natural foods = triangles; human derived foods = squares). Available protein contribution is the implicit axis. Each point represents an energy mixture, e.g., the labeled food point represents a naturally derived food consumed by the subject (pine nuts) that supplies 8% metabolizable energy from carbohydrates, 85% from fat and 7% from available protein. The dashed line shows the linear regression of natural food composition (y = −0.57x +53.54, R^2^ = 0.61), and the dotted line shows the linear regression of diet composition (y = −1.06x +88.94, R^2^ = 0.91). Variation in the diet across days was relatively stable for available protein (AP), shown by the limited scatter around the dotted line and a slope not significantly different from one (F = 0.95, df = 28, p-value = 0.34), whereas the proportion of fat (F) and carbohydrate (C) in the diet varied more across days (shown by greater scatter along the dotted line). The relative contribution of AP, C and F to the diet contrasted with the distribution of macronutrients in natural foods, which varied most in their balance of AP to C.

**Figure 2:**
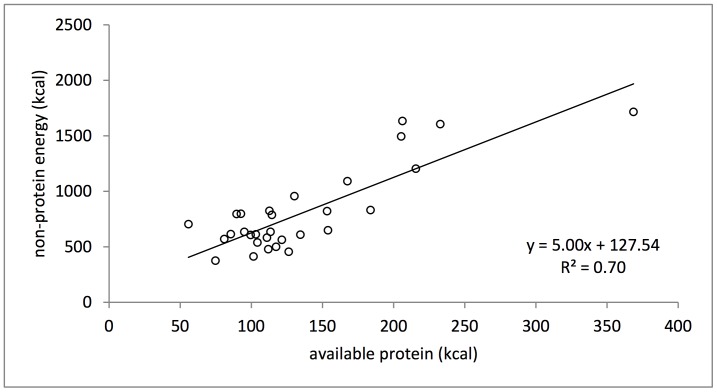
Daily intake of non-protein energy vs. available protein for days 1–30.

**Figure 3:**
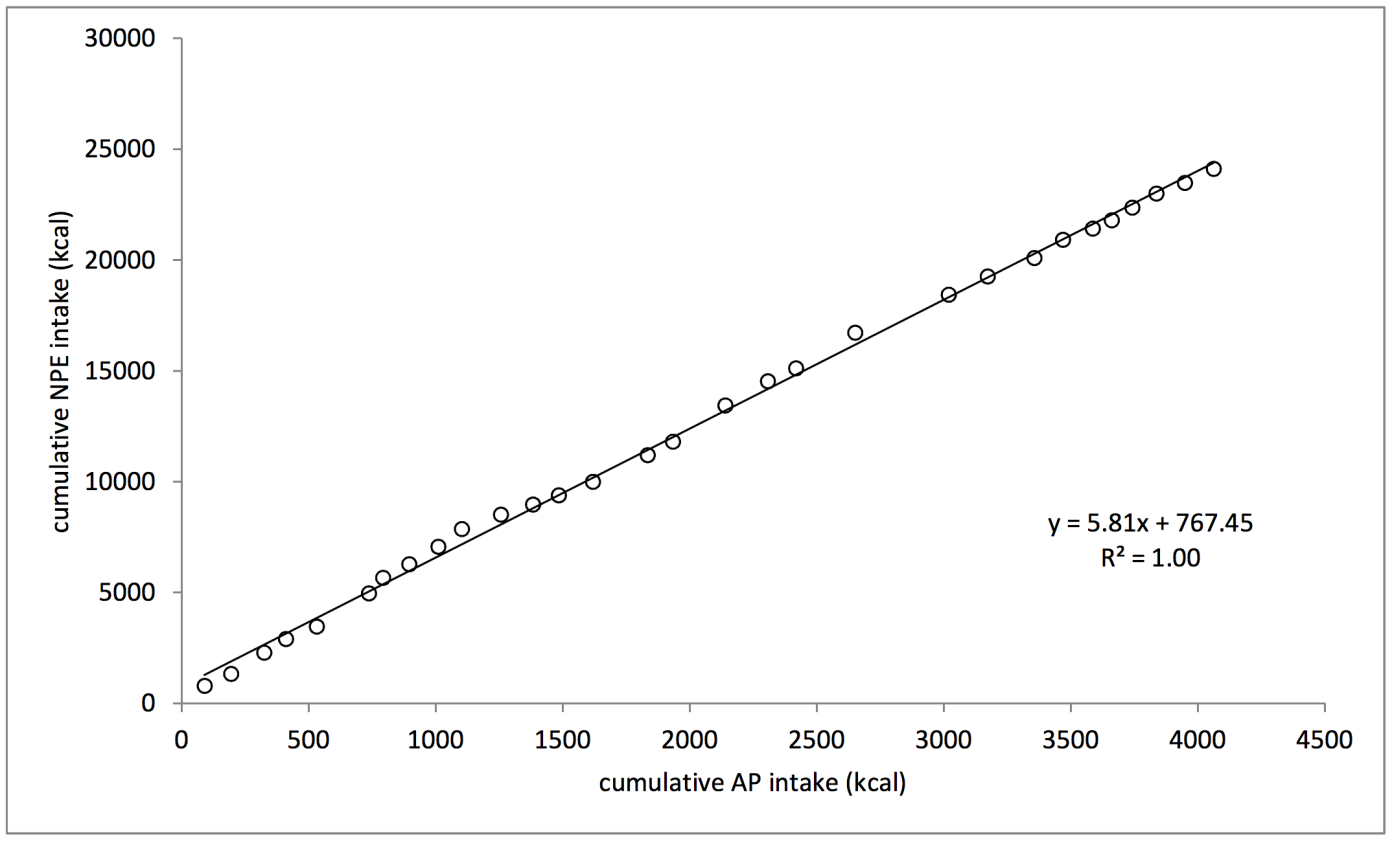
Cumulative intake of NPE vs. AP across 30 days.

The focal subject’s average daily energy intake was 940±426 kcal/day. She maintained a balance of non-protein energy (NPE) to available protein (AP) in her diet through relatively constant proportional intake of AP, while using fats and carbohydrates as interchangeable sources of NPE (Fig. 1). The least variable nutrient intake observed for the subject was her balanced intake of AP to NPE (CV = 0.31, R^2^ = 0.70). Across the 30 days, she maintained an average daily intake of 5∶1 kcal NPE to AP (Fig. 2). The long-term cumulative balance of daily food selection was highly consistent, showing that the subject regulated her intake of macronutrients over a single daily observation period, i.e., from dawn to dusk (R^2^ = 1.00) (Fig. 3).

## Discussion

The results of this study demonstrate a remarkable consistency in our focal subject’s intake of non-protein energy to available protein (Fig. 2) amid fluctuation in the intake of other nutrients and non-nutrient items. To maintain this balance, she ingested fats and carbohydrates as interchangeable sources of energy while maintaining a consistent relative intake of available protein (Fig. 1). The relative contribution of available protein, digestible carbohydrates and fats to the diet did not match the distribution of these macronutrients in natural foods, which varied most in their balance of available protein to digestible carbohydrates (Fig. 1). This demonstrates that our focal subject was able to regulate to a macronutritional intake target within this anthropogenically altered habitat.

The long-term integrated dietary macronutrient balance associated with daily food selection for the focal subject was notably constant within a 24-hour period (Fig. 3). This suggests that the observation period represented a time of stable nutrient demands in the life of this adult female, who was neither pregnant nor lactating at the time of the study. In contrast, systematic changes have been observed during reproduction and other developmental change in some other animals [46]. The results of this study demonstrate the value of estimating daily nutrient intake on an individual level across days in order to more accurately reveal patterns of dietary regulation over time. While we cannot necessarily generalize our results to all chacma baboons or even to this entire population, our continued observations on this troop suggest that the diet of the study subject does not differ from that of other baboons in the Tokai Forest, and that data from other females in this study troop would be similar to those from the focal subject in their broad patterns.

Other field applications of the geometric framework of nutrition to primate diets have considered intakes by multiple individuals over periods of one day or less with constraints on food availability [11]–[13]. When prevented from balancing protein to non-protein energy intake due to food availability constraints, humans and wild spider monkeys (*Ateles chamek*) prioritize intake of protein [11], [13], while wild mountain gorillas (*Gorilla beringei*) prioritize intake of non-protein energy [12]. Here we could not assess whether our focal subject prioritized intake of non-protein energy or available protein when unable to achieve the target macronutrient balance, as over consecutive days she was able to combine available foods to achieve a near-balanced diet on all observation days (Fig. 2). In order to assess this, we would need data from circumstances in which available foods constrain the subject from ingesting a balanced diet, for example across seasons [11], [12].

Like other baboons, chacmas are omnivorous [21], [26], and this dietary classification is reflected in the balance of nutrients ingested by our focal subject compared to that of other primates. Spider monkeys, for example, are frugivores and non-protein energy should thus be more abundant in the diet due to a focus on fruit sources [47], [48]. These high levels of non-protein energy intake are reflected in spider monkey diets, which are characterized by a balance of 8∶1 kcal non-protein energy to available protein [17]. Spider monkeys are probably constrained in their regulatory balance by the digestive limitations of rapid gut transit times, while high protein but fibrous and therefore difficult to digest foods are available to them mainly in the form of leaves [12], [48]. By contrast, mountain gorillas are seasonal in their frugivory [41], [49], with leaves comprising a greater proportion of the diet overall. Correspondingly, mountain gorillas maintain a balance of 3∶1 kcal non-protein energy to available protein during fruiting periods and 2∶1 kcal non-protein energy to available protein when fruits are less seasonally abundant, and thus their regulatory balance reflects differences in food availability [12]. As we would expect from a generalist omnivore incorporating a greater diversity of food resources in the diet, our focal subject’s non-protein energy to available protein balance, at 5∶1 kcal, is intermediate between that of a frugivorous and a folivorous primate.

The average daily energy intake for a chacma baboon female in captivity was found by Bielert and Busse [19] to be 51 kcal per kg of body weight. Because our subject was a wild baboon, she likely expends more energy and should thus have a higher daily energy intake compared to a captive animal [50]. Using the captive baboon estimate from Bielert and Busse [19] and an average wild chacma baboon female weight of 15.5 kg [51], [52], we would predict a daily energy intake of 791 kcal for our focal subject, which is indeed lower than her actual intake of 940 kcal/day. There are no available estimates of average daily energy intake for other wild chacma baboons, but if we use Stacey’s [53] estimate of 92 kcal per kg of body weight for wild yellow baboons (*Papio hamadryas cynocephalus*), we would predict our subject to have an average daily intake of 1426 kcal. Our subject’s actual daily energy intake (940 kcal/day) was lower than this estimate. This is not surprising given that baboons in Tokai have shorter daily travel distances, smaller home ranges and lower overall travel time than other baboons due to high concentrations of food resources and abundant sleeping sites [28], [54].

Despite the close proximity of the study troop to multiple sources of human-derived foods within their home range, such foods contributed a relatively small portion of the diet by dry mass, and also contributed little to the focal subject’s total energy intake. Qualitative observations suggest that other baboons in this population, such as high ranking males, participate more in raiding and consumption of human-derived foods compared to the study subject and other females. As shown in Figure 1, the human-derived foods that were consumed were characterized by comparatively high levels of one or two macronutrients and thus provided a relatively unbalanced diet. Moreover, the human-derived foods consumed by STE may have contained lower levels of macronutrients than estimated by their fresh USDA counterparts, given that they were mainly consumed from sources such as waste bins and perhaps had undergone decomposition and degradation.

Notably, almost half of the subject’s energy intake derived from exotic food items such as pine nuts (*Pinus pinea*) and acorns (*Quercus* sp.). Dandelions (*Tolpis capensis*), native to South Africa but often found in disturbed areas [55], were also a major source of calories (about 19% of energy intake). The large contribution of *Pinus* and *Quercus* to the diet highlights the ecological flexibility of these opportunistic omnivores in taking advantage of the high fat content of the seeds of these two non-native species. This shift in diet must have occurred at some point after the initial establishment of the plantation in the late 19^th^ century. This heavy focus on exotic items for energy may also relate to their greater abundance, as they have more standing biomass and higher seed production than the native vegetation in this area [27], [32].

Most exotic species in Tokai consume more water and soil nutrients compared to the native oligotrophic and mesotrophic fynbos vegetation [33], and for this and other socio-political reasons the Tokai plantation is currently being commercially harvested so that the indigenous fynbos vegetation may be restored [28]. These landscape transformations will, at the very least, dramatically reduce the availability of the pine nuts and acorns that comprised 44% of the energy intake for our subject. While some baboon troops in the Cape Peninsula subsist almost exclusively on fynbos vegetation, supplemented by marine resources [32], the energetically rich exotic resources and human-derived foods found in Tokai allow a far higher population density and shorter daily path lengths compared to some other troops [56]. The conversion to fynbos will thus likely include a transition period for the Tokai baboons as they behaviorally and demographically adjust to the new patterns of food availability and associated lower carrying capacity of the area. This transition period may include an even greater focus on human-derived foods, thereby increasing levels of baboon-human conflict, unless steps are taken to dramatically reduce baboon access to human foods. Future research on nutritional goals and nutrient regulation in this and other baboon populations will be crucial in managing and conserving populations that are experiencing similar anthropogenic habitat change.

Primate diets are typically characterized by macronutrient-dominated diet regulation. However, we have as yet been unable to adequately test the relative roles of environment and phylogeny in shaping nutrient regulation in primates due to a lack of appropriate data sets. Baboons of the genus *Papio* are a potentially excellent model species with which to attempt to unravel these effects, as they are closely related yet occupy an exceptionally wide array of habitat types. The data presented here are crucial in moving towards a more comprehensive comparison of diet composition and nutrient regulation in baboons across habitat types. This information will lead to a greater understanding of the relative roles of the environment and phylogenetic history in shaping nutrient regulation among primates as a whole.
